# Platelet-rich plasma (PRP) in osteoarthritis (OA) knee: Correct dose critical for long term clinical efficacy

**DOI:** 10.1038/s41598-021-83025-2

**Published:** 2021-02-17

**Authors:** Himanshu Bansal, Jerry Leon, Jeremy L. Pont, David A. Wilson, Anupama Bansal, Diwaker Agarwal, Iustin Preoteasa

**Affiliations:** 1 Mother Cell Spinal Injury and Stem Cell Research, Anupam Hospital, Rudrapur, Uttarakhand India; 2 PMR Advance Health Institute Mayaguez, Puerto Rico, USA; 3 Pheonix Helse, Lillesand, Norway; 4 Mercy Medical Centre, Roseburg, OR USA; 5 Alpha Medica Stem Clinic, Voineşti, Romania

**Keywords:** Biological techniques, Medical research

## Abstract

Despite encouraging results reported with regards to Platelet-rich plasma (PRP) application in osteoarthritis (OA) knee, still critical issues like conclusive structural evidence of its efficacy, standard dose and good manual method of preparation to obtain high yield remains unanswered. Present study is an attempt to optimise the dose and concentration of therapeutic PRP and its correlation with structural, physiologic efficacy with a new manual method of PRP preparation. A total of one hundred and fifty patients were randomized to receive either PRP (10 billion platelets) or hyaluronic acid (HA; 4 ml; 75 patients in each group) and followed up till 1 year. An addition of filtration step with 1 µm filter in manual PRP processing improved platelet recovery upto 90%. Significant improvements in WOMAC (51.94 ± 7.35 vs. 57.33 ± 8.92; *P* < 0.001), IKDC scores (62.8 ± 6.24 vs 52.7 ± 6.39; *P* < 0.001), 6-min pain free walking distance (+ 120 vs. + 4; *P* < 0.001) persisted in PRP compared to HA group at 1 year. Significant decline IL-6 and TNF-α levels observed in PRP group (*P* < 0.05) compared to HA at 1 month. Study demonstrated that an absolute count of 10 billion platelets is crucial in a PRP formulation to have long sustained chondroprotective effect upto one year in moderate knee OA.

## Introduction

Osteoarthritis (OA) is a leading cause of severe long-term pain and disability affecting approximately 10% of the global population^1^. Regenerative solutions and new tissue- engineering based strategies are promising for treatment of moderate OA^2,3^. The research for treatment of knee OA with PRP is promising^4–6^, however there is a lack of consensus regarding the preparation of standardized dosing with an appropriate absolute number of platelets and concentration. Studies often report PRP preparations taken from between 20 to100 ml of blood, with a concentration of 2–10 × 10^6^ platelet/µl^4–6^.

Most manual methods fail to provide a high yield and often have variable concentrations ranging two to four times of physiological count^7^. Alternatively, clinicians may be reliant on expensive kits, ranging from between $150–$250 per treatment^7^. In order to have a high consistent platelet yield we designed a filtration-based manual method. This prospective randomized controlled study was primarily aimed at standardizing the ideal PRP dosage and concentration, and to assess the subjective, structural and physiological efficacy of PRP in OA knee.

## Results

### Patients screened for clinical trial

**Figure 1:**
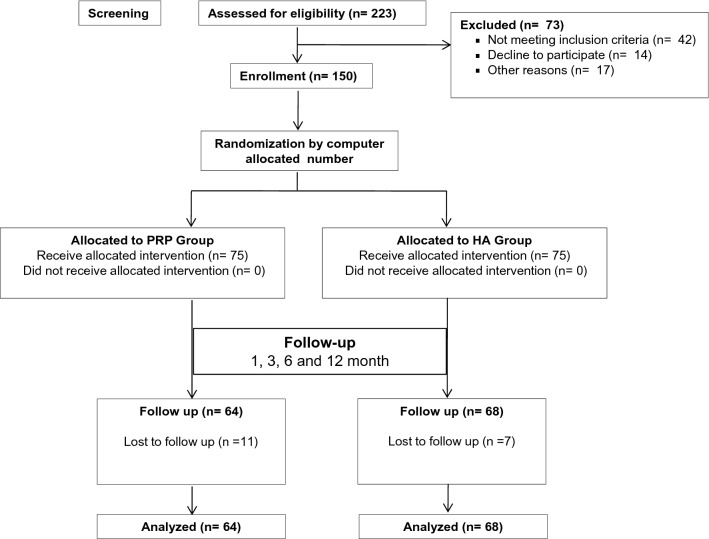
Flowchart of the clinical trial: Screening, assessment, treatment allocation and follow-up of patients with OA. *n* number of patients.

One hundred and fifty randomized subjects were recruited and treated with PRP or HA (75 patients respectively) during 2014–2017.A total of 64 patients in the PRP group and 68 patients in the control (HA) group were followed up till 2018 (Fig. 1). There were no significant differences in clinical characteristics between the groups (Table1).

**Table 1:** Baseline clinical characteristics of patients with OA knee treated with PRP or HA.

Baseline characteristics	PRP (*n* = 64)	HA (*n* = 68)
**Demographic characteristics**		
Age (years; mean)	64.4 (52–74)	65.8(54–73)
Sex (*n* = male)	39	42
Weight (kg; mean)	70.6	71.2
Height (cm; mean)	168.4	167.8
Right knee (*n*)	36	33
IKDC score	53.6	54.2
WOMAC score (total)	52–66 Mean = 54.97	50–68 Mean = 53.56
6MWD range (mean)	1224–1488 (1320)	1190–1520 (1386)
Cartilage thickness (mm; MRI)	4.48–4.98	4.43–5.00
Range (mean)	(4.61)	(4.64)
JSW (mm)	3.42–4.68	3.48–4.72
Range (Mean)	(3.81)	(3.78)
**Osteophyte score (4 grade) no. (%)**		
0	2 (3.1)	3 (4.4)
1	26 (40.6)	30 (44.1)
2	33 (51.5)	32 (47.1)
3	3 (4.68)	3 (4.4)
**Kellgren and Lawrence score (5 grades) number (%)**		
0	0 (0)	0 (0)
1	2 (3.1)	3 (4.4)
2	8 (12.5)	9 (13.2)
3	54 (84.3)	56 (82.3)
4	0(0)	0 (0)

*6MWD* 6-min walking distance, *PRP* Platelet-rich plasma, *HA* Hyaluronic acid, *IKDC* International Knee Documentation Committee, *JSW* joint space width, *WOMAC* Western Ontario and McMaster Universities Osteoarthritis Index, *n* number of patients.

### PRP analysis

The baseline platelet count ranged from 1.91 to 3.25 × 10^5^ platelet/µl (mean 2.3 × 10^5^ platelet/µl). The PRP concentrate had a platelet count ranging from 12.68 to 16.2 × 10^5^ platelet/µl (mean 14.38 ± 1.76 × 10^5^ platelet/µl) with a recovery of 90% (87.4–92.6%).The total platelet count in fused ranged from 10.14 to 10.83 billion (10.45 ± 0.46) in 8 ml of PRP. The total leukocyte count was zero in our PRP analysis. The PDGF concentration in the PRP ranged from 50,246 to 74,938 pg/ml (63,668 ± 12,968 pg/ml) and VEGF from 1348 to 2429 pg/ml (1788 ± 1245 pg/ml).

### Patient evaluation and pain score

**Figure 2:**
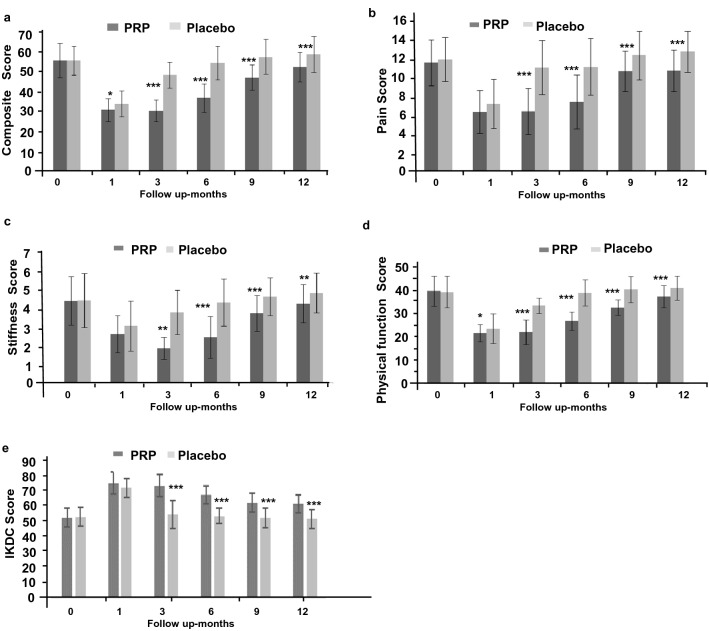
Graphical representation of Western Ontario and McMaster Universities Osteoarthritis Index (WOMAC) score of patients in PRP and control groups over 1 Year (**a**) composite scores, (**b**) pain score, (**c**) stiffness score, (**d**) physical function score (**e**) Graphical representation of IKDC score.

Symptomatic outcome measure WOMAC composite scores showed significant improvement from baseline in both PRP (*P* < 0.001) and HA groups (*P* < 0.001) at one month. Although PRP group had better scores than HA at one month but were statistically insignificant (*P* > 0.05). This improvement declined with time and was more profound in HA group. The scores in HA group were marginally better but insignificant at 3 months, reached to baseline levels at 6 months and further dropped inferior at 9 months and 12 months’ time frame. Whereas PRP group reported significantly better scores during follow-up until one year (*P* < 0.05: Table 2; Fig. 2a–d). Intergroup comparison indicated significant better composite scores in PRP group compared to HA group at 3 (*P* < 0.001), 6 (*P* < 0.001) and 9 months (*P* < 0.01) and 1 year (*P* < 0.001) respectively.

**Table 2:** Statistical analysis of WOMAC scores of patients in PRP (n = 64) and placebo HA (n = 68) groups over 1 Year of follow-up.

Womac composite score			
Month	PRP vs HA	PRP vs PRP	HA vs HA
0	0.860		
1	0.038	< 0.001	< 0.001
3	< 0.001	< 0.001	< 0.01
6	< 0.001	< 0.001	0.817
9	< 0.001	< 0.01	0.445
12	< 0.001	0.042	0.193

Pain score			
Month	PRP vs HA	PRP vs PRP	HA vs HA
0	0.371		
1	0.108	< 0.001	< 0.001
3	< 0.001	< 0.001	0.052
6	< 0.001	< 0.001	0.14
9	< 0.01	0.031	0.887
12	< 0.01	0.043	0.525

Stiffness score			
Month	PRP vs HA	PRP 0 vs PRP	HA 0 vs HA
0	0.913		
1	0.054	< 0.001	< 0.001
3	< 0.01	< 0.001	< 0.01
6	< 0.001	< 0.001	0.61
9	< 0.001	< 0.01	0.327
12	< 0.001	0.044	0.063

Physical function score			
Month	PRP vs HA	PRP vs PRP	HA vs HA
0	0.738		
1	< 0.05	< 0.001	< 0.001
3	< 0.001	< 0.001	< 0.01
6	< 0.001	< 0.001	0.929
9	< 0.001	< 0.01	0.314
12	< 0.001	< 0.036	0.101

*WOMAC* Western Ontario and McMaster Universities Arthritis Index: scores are in mean ± SD (95% CI; confidence interval).

The WOMAC sub-score for pain declined significantly in one month (*P* < 0.001) followed by worsening of scores in subsequent follow-up and finally to reach inferior to baseline at 12-month (Table 2, Fig. 2b–d) in HA group. Whereas, the pain sub scores were significantly better up to 12 months (*P* < 0.05; Table 2, Fig. 2b) in PRP group. Intergroup comparison indicated significant better pain scores in PRP group compared to HA group at 3, 6, 9 and 12 month (Table2; Fig. 2b). The trends of WOMAC stiffness and physical function were similar to composite score and pain pattern (Table 2; Fig. 2c–d).

The PRP group demonstrated improvement in IKDC scores (*P* < 0.001) at one-month which slightly decreased but remained significantly better than baseline at all time frame unto one year (*P* < 0.01; Table 3; Fig. 2e). In the HA group, there was significant improvement (*P* < 0.001) at one month but this gradually deteriorated at 3, 6, and 9 and 12 month follow-up with scores below base line at one year. The difference between PRP and HA was insignificant at one month (*P* > 0.05) but significant at all other time frame until one year *(P* < 0.001; Table 3; Fig. 2e).

**Table 3:** IKDC scores of patients in PRP and HA groups over 1 Year.

Duration	Treatment	IKDC	*P-*value baseline vs time point
Baseline	PRP	53.6 (± 6.34)	
	HA	54.2 (± 6.28)	
*P* value PRP vs placebo at baseline			*P* > 0.05
1 month	PRP	76.9 (± 7.43)	*P* < 0.001
	HA	73.75 (± 6.21)	*P* < 0.001
*P* value PRP vs placebo at 1 month			*P* > 0.05
3 months	PRP	75.20 (± 7.55)	*P* < 0.001
	HA	55.82 (± 9.33)	*P* > 0.05
*P* value PRP vs placebo at 3 month			*P* < 0.001
6 months	PRP	68.9 (± 6.21)	*P* < 0.001
	HA	54.8 (± 5.17)	*P* > 0.05
*P* value PRP vs placebo at 6 month			*P* < 0.001
9 months	PRP	63.5 (± 6.38)	*P* < 0.001
	HA	53.4 (± 6.78)	*P* > 0.05
*P* value PRP vs placebo at 9 month			*P* < 0.001
12 months	PRP	62.8 (± 6.24)	*P* < 0.01
	HA	52.7 (± 6.39)	*P* > 0.05
*P* value PRP vs placebo at 1 year			*P* < 0.001

*DC* International Knee Documentation Committee, *PRP* platelet-rich plasma, *HA* Hyaluronic acid.

We observed significant improvement (*P* < 0.001) in pain-free distance covered during a 6MWD at 1 and 3 months in both the groups. However, the control (HA) group could not sustain the improvement at 6, 9 and 12 months (*P* > 0.05; Table 4). Significant improvement (*P* < 0.05) at month was maintained among PRP group as compared to HA in all time frame upto 1 year (*P* < 0.001; Table 4).We observed that 24% of patients in the PRP group showed an improvement when covering 100 ft distance at three months compared to 11% in the control group (Table 4).

**Table 4:** Pain-free distance covered during 6MWD test and Joints width in PRP and HA groups over 1 Year.

	Time point	PRP group	HA group	*P-*value PRP vs HA
Pain-free distance covered during 6MWD	Baseline	1320	1336	*P* > 0.05
	1 month	+ 146	+ 122	*P* < 0.05
	*P*-value at 1 month	*P* < 0.001	*P* < 0.001	
	3 months	+ 140	+ 48	*P* < 0.001
	*P*-value at 3 month	*P* < 0.001	*P* < 0.001	
	6 months	+ 136	+ 35	*P* < 0.001
	*P*-value at 6 month	*P* < 0.05	*P* > 0.05	
	9 months	+ 125	+ 8	*P* < 0.001
	*P*-value at 9 month	*P* < 0.05	*P* > 0.05	
	1 year	+ 120	+ 4	*P* < 0.001
	*P*-value at 1 year	*P* < 0.001	*P* > 0.05	
Joint space width as measured on standing X-ray	Baseline	3.81	3.78	*P* > 0.05
	1 year	3.77	3.68	*P* > 0.05
Unchanged cartilage thickness in MRI number of patients (%)	1 year	53 (82.8)	42 (61.7)	*P* < 0.05

*6MWD* 6-min walking distance, *PRP* Platelet-rich plasma, *HA* Hyaluronic acid, *n* number of patients.

At one-year rescue medication was required at least once a week by 24 (37.5%) patients in the PRP group, and 36 (52.9%) in the control group (*P* < 0.001). Furthermore, there was a 26% reduction in the use of paracetamol in the PRP group, as compared to control. X-ray evaluation demonstrated that there was no increase in JSW, rather both the groups had deterioration (*P* < 0.05; Table 4), but it was better maintained in the PRP group though insignificant difference (*P* > 0.05; Table 4). Intra class correlation (ICCTs) was insignificant at one year between PRP and control group for JSW*.* Decrease in JSW (≥ 0.5 mm) was observed in 3 (4%) patients in PRP and 8 (10.6%) patients in control group.

**Figure 3:**
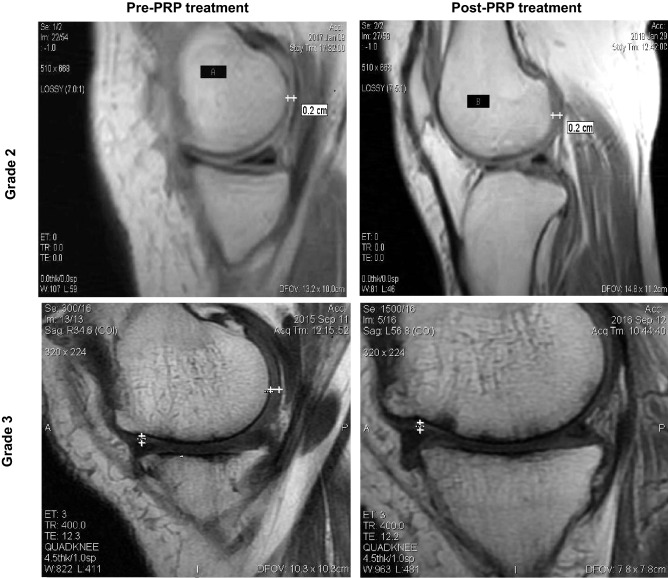
Evaluation of articular cartilage thickness using MRI after one year of PRP treatment in patient with grade 2 and grade 3 OA knee.

Increase in cartilage thickness was not observed on MRI in either group (Fig. 3). In the PRP group, it remained unchanged in 53 (82.8%) patients at one year as compared to 42 (61.7%) patients in control (*P* < 0.05). Sixteen (23.5%) patients lost thickness in control group as compared to 11 (17.1%) patients in PRP group at one-year follow-up (*P* > 0.05; Table 4).

### Cytokine analysis

**Figure 4:**
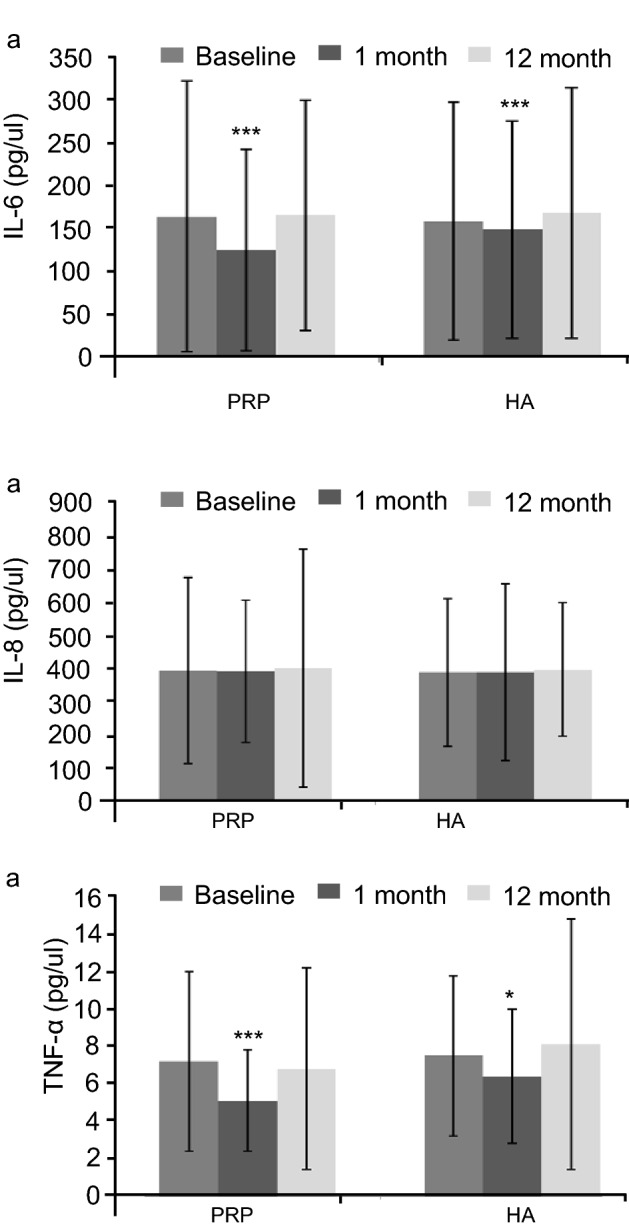
Level of cytokines IL6, IL8 and TNF-α in PRP and HA group.

All the patients had high level of IL-6, IL-8 and TNF-α in the synovial fluid at baseline. We observed significant difference in level of IL-6 (124.2 ± 117.3 pg/mL vs. 148.4 ± 126.6 pg/mL; *P* < 0.05) and TNF-α (5.1 ± 2.7 pg/mL vs. 6.4 ± 3.6 pg/mL; *P* < 0.05) among PRP and control group at 1 months (Fig. 4). The level of IL6 and TNF-α was correlated with WOMAC score at 1 month in both PRP and HA treated group. We did not find any significant difference in level of IL-8 among PRP and HA levels (*P* > 0.05; Fig. 4, Supplementary Table 3).

### Adverse effects

Both groups had equal numbers of patients with mild transient adverse events. Pain, stiffness and synovitis were the most common complaints (Supplementary Table 1). There were no permanent adverse effects to any participants.

## Discussion

Recently PRP has been extensively explored as a chondro-protective treatment for symptomatic knee OA^8^. Our study demonstrated that a dose of 10 billion platelets in 8 ml volume of PRP improves functional outcomes and protects the articular cartilage from further wear and tear in patients with knee OA.

The results of WOMAC, IKDC and 6MWD improved significantly in the first-month itself with PRP injection and despite slight worsening 3, 6, 9 and 12-month follow-up, were still significantly better than the HA group. In the HA group, improvements noted at 1 month were not present at 3, 6, 9 and 12-month follow-up. The overall difference between the PRP and control groups at one year strongly suggests the efficacy of PRP as a treatment for OA. As expected, the study demonstrated no structural efficacy of PRP unlike cellular therapy^9^. We observed, however, that PRP had a chondro-protective structural benefit in terms of better maintenance of the JSW and cartilage thickness as an outcome measure.

Our results correlates well with earlier studies^10–12^ although, direct comparison is difficult because of differences in PRP processing, the dose (quantity and concentration of platelets), and no standard structural efficacy criteria. Despite all these odds a recent meta-analysis of 30 RCT demonstrated best overall outcome in patients treated with PRP as compared to control, HA or steroids at 3, 6, 12 months follow up intervals^13^ which correlated well with our results. In a randomized study with PRP and HA treatment the IKDC score was significantly higher in the PRP group at 24 and 52 weeks (*P* < 0.01)^14^. Similarly, significant improvements were demonstrated in our PRP group for IKDC scores (*P* < 0.05) at 12 weeks^15^. Another study reported improvement in IKDC scores despite the absolute number of platelets injected being very low at 6.5 million per knee^16^. A single dose of PRP in 22 patients (ages of 30–70 years) with early knee OA improved pain function scores at 6 months and 1 year. No visible changes on MRI were found in at least 73% of the patients at 1 year^17^. Our study however included elderly and moderate OA patients with a positive outcome.

A significant improvement in WOMAC scores within 2–3 weeks with worsening at 6-months was reported after treatment with two injections of WBC-filtered PRP with an average absolute count of 23.85 billion platelets injected per knee^17^. Our study shows maintained effectiveness at one year after a single injection. WOMAC and IKDC scores were previously shown to be significantly better with PRP than HA injections (*P* < 0.001) in four randomized controlled trials^18^.

We also evaluated the clinical correlation of the pro inflammatory cytokines IL-6 and TNF-α with WOMAC scores at 3 months in the PRP and HA groups. Our data suggests that decrease in inflammatory cytokines in the knee with subsequent clinical improvement in patient-reported outcomes at 3 months are time dependent. We did not find any statistical difference in levels of cytokines between groups. Cytokine levels were correlated with the degree of pain as previous study^19^.

Minimal manipulated processing and optimal dose of platelets is very crucial in PRP to obtain clinically effective results^20,21^. Obtaining PRP is often expensive and could be restrictive in developing countries with inadequacy of resources. Most of the “manual” methods have drawback as many platelets are lost if not filtered. Our novel methodology with indigenous one-micron filters recovered them from the PPP improving yield upto 92%.

Whether presence of leukocytes in PRP preparation can damage cartilage is highly debated^22^. Studies have shown that leucocytes in PRP can damage cartilage whereas leukocyte-poor PRP, promotes chondrogenesis in vivo^22^ and better functional outcomes^23^.

The growth factors secreted by the platelets stimulate the proliferation of chondrocytes and mesenchymal stem cells thereby assisting in synthesis of type II collagen^21^. Suppression of mediators such as IL-1 interaction^24^ with nociceptors^22^ brings inflammatory and analgesic effects.

Significant clinical effects were observed in control (HA) group upto 1 month were due to the lubricating and shock-absorbing properties of HA^25^.

Application of HA in moderate OA has been reported to decrease in the average number of opioid prescriptions as well as overall new prescriptions^26^, better maintenance of medial and lateral joint space areas^27^, delay in total knee replacement surgery^28,29^ in moderate OA. However American academy of orthopedic surgeons AAOS (2013) and American college of Rheumatology ACR (2020) recommended against use of HA and considered that it is not medically necessary for the treatment of pain^30^. Heterogeneous trial results conflicting conclusions, and flaws in interpreting data make literature interpretations very confusing. However majority have reported clinically important reductions in pain and excellent safety profile^31^.

We used inactivated PRP as it increases proliferation of the mesenchymal stem cells fivefold^32^, improves cartilage, and aids in bone formation. Activated PRP may inhibit chondrogenesis and osteogenesis in vivo^33^. Need of activation of PRP prior to injection is an issue on ongoing Debate. Several studies have reported that activation of PRP before joint injection ensure that signaling elements are released during fibrin retraction and fibrinolysis^34^ hence better results on degenerative cartilage lesions^20^.

MRI interpretations methodology of our study for minor improvements in cartilage is too small to be consistently and reliably picked up. In present study MRI evaluation should have beenwithT2wetmaps. Mere addressing the cartilage loss would be unlikely to succeed in OA treatment if not focused on correcting the abnormal mechanics and ligament laxity^35^. Approximately 50%of the patients with radiological changes of OA are asymptomatic because articular cartilage is not innervated^36^ hence in many situations despite chondroprotection provided by PRP pain may persist.

The strengths of our study are the structural and physiological evaluation, standardized PRP processing with little variation, and a very high level of consistency in absolute platelet counts (≥ 10 billion) in 8 ml which we hope will help in standardizing the dosage for treatment. “The major limitation of our study was the absence of a true control group using saline. The study did not address implication of PRP in advanced OA. Besides small sample size and assessment in limited time frame, the study was limited by variable doses (different combination of absolute number and concentration of platelets in PRP) evaluation.

Our study provides evidence that clinical outcome does not only depend upon the concentration, but also on the absolute platelet count. We delivered a standardized PRP dose with little variation. Injecting 8 ml PRP in joint space through supra lateral approach does not produce any distension or swelling and is safe as knee joint has large volume and surface area^37^. Critical dose is important for sustained therapeutic effect. We have observed sustained therapeutic benefit with dose of 10 billion platelets in 8 ml volume of PRP. It can be hypothesized that higher platelet counts will ultimately lead to high growth factors release hence generate better outcome. However further studies are needed to evaluate if still higher doses (more than 10 billion) are more beneficial or counterproductive and similarly higher concentration (less volume of PRP with 10 billion platelets) would yield the same result.

## Conclusion

Application of PRP with absolute counts of 10 billion platelets in a volume of 8 ml provides significant potential chondro-protection and alleviates symptoms compared to control in knee OA.

## Material and methods

### Eligibility and patient selection

This trial was ethically approved by the Institutional Committee for Stem Cell Research and Therapy, Anupam Hospital, Uttarakhand, India. The trial is compliant with consolidated standards of reporting trials (CONSORT). Informed prior consent was obtained from all the patients. The criteria for patients selection was age ≥ 50 years with symptomatic primary knee OA (Supplementary Table 2). The more painful knee was considered in cases where the patient had bilateral OA. (Clinicaltrials.gov-NCT04198467; Date of registration 13/12/2019; ClinicalTrial.gov under URL: https://clinicaltrials.gov/ct2/show/NCT04198467).

### Preparation of PRP

A blood sample (60 ml) with 10% ACD solution was drawn and centrifuged at 600×*g* for 10 min before the plasma fraction was collected. The plasma fraction was centrifuged at 4000×*g* for 15 mins^9^. Supernatant platelet poor plasma (PPP) was then removed, leaving 3 ml PRP^9^. The PPP was passed through a one-micron special flush-back filter (Alpha Corpuscle, New Delhi, India) so that all the platelets present in PPP fraction were trapped in the filter before being flushed back with 5 ml of PPP to retrieve the captured platelets then mixed with the previous 3 ml PRP. The mixture was passed through a WBC filter (Terumo Imuguard, CO, USA) to remove the leukocytes. Platelet counts were adjusted to 10 billion in 8 ml of volume. We used inactivated PRP. The product was analyzed for total leukocyte and platelet counts. Platelet counts were adjusted to 10 billion in 8 ml of volume by diluting it with PPP in patients having high baseline values hence yielding higher counts. Five samples were selected randomly to assess platelet-derived growth factors (PDGF) and vascular endothelial growth factors (VEGF) by ELISA.

### Control

Four milliliters of high-molecular-weight hyaluronic acid (HA: Brand name: Monovisc from Anika Therapeutics, Inc., MA, USA) with a concentration of 22 mg/ml was selected as treatment for the control group.

### Study design

This prospective, double-blinded, randomized control (parallel designed with allocation 1:1 ratio), 12-month, study of 150 outpatients was conducted following the 1964 Declaration of Helsinki^38^. All methods were carried out in accordance with relevant guide lines and regulations. Patients were randomly selected based on a computer-generated number to receive one indistinguishable injection of either PRP or HA. Followings kind is infection of the knee joint, PRP (8 ml; we used inactivated PRP) or HA was injected into the joint space through supralteral approach. Participant patients and the physician who assessed the outcome were blinded to treatment arm. Patients were advised to continue with physiotherapy and knee exercises. Paracetamol, to a maximum of up to one gram three times a day, was prescribed as a rescue drug.

### Cytokine analysis

One ml of synovial fluid was aspirated from patients and evaluated for pro-inflammatory cytokines IL-6, IL-8and TNF-α (Quantikine ELISA kits, R & D system, Canada) using ELISA at 0, 1 and 12 months.

### Study assessments

Patients were assessed by WOMAC^39^, IKDC scores^40^ and 6-min walking distance (6MWD) at 0, 1, 3, 6, and 12 months. The structural efficacy was evaluated by joint space width (JSW) on X-ray and articular cartilage thickness on MRI at baseline and at 12 months by two experienced radiologists with 5% disagreement. Assessors were blinded to the treatment of PRP or control. The joint space width at the narrowest point of joint^41^ and Kellgren and Lawrence grade^42^ were assessed. MRI evaluation was performed as previously described^9,43^. The patients were initially followed up once weekly during the first month then once a month for the remaining 11 months for reporting of adverse effects, and biochemical and hematological analysis.

### Statistical analysis

All statistical analyses were done as an intention to treat (ITT). Two tailed testing was performed using SPSS 20.0 statistical software package (SPSS Inc., Chicago, USA). The Kolmogorov–Smirnov test was performed to determine the normal distribution of continuous variables^44^. The repeated variant analysis was performed to assess the time variance of the variables. Statistical significance was *P* < 0.05.

## Supplementary Information

Supplementary Information.
