# Author Correction: Platelet‑rich plasma (PRP) in osteoarthritis (OA) knee: Correct dose critical for long term clinical efficacy

**DOI:** 10.1038/s41598-021-98365-2

**Published:** 2021-09-14

**Authors:** Himanshu Bansal, Jerry Leon, Jeremy L. Pont, David A. Wilson, Anupama Bansal, Diwaker Agarwal, Iustin Preoteasa

**Affiliations:** 1 Mother Cell Spinal Injury and Stem Cell Research, Anupam Hospital, Rudrapur, Uttarakhand India; 2 PMR Advance Health Institute Mayaguez, Puerto Rico, USA; 3 Pheonix Helse, Lillesand, Norway; 4 Mercy Medical Centre, Roseburg, OR USA; 5 Alpha Medica Stem Clinic, Voineşti, Romania

Correction to: *Scientific Reports* 10.1038/s41598-021-83025-2, published online 17 February 2021

The original version of this Article contained errors in Figure 3 where the incorrect MRI images were used. The original Figure 3 and accompanying legend appear below.

**Figure 3:**
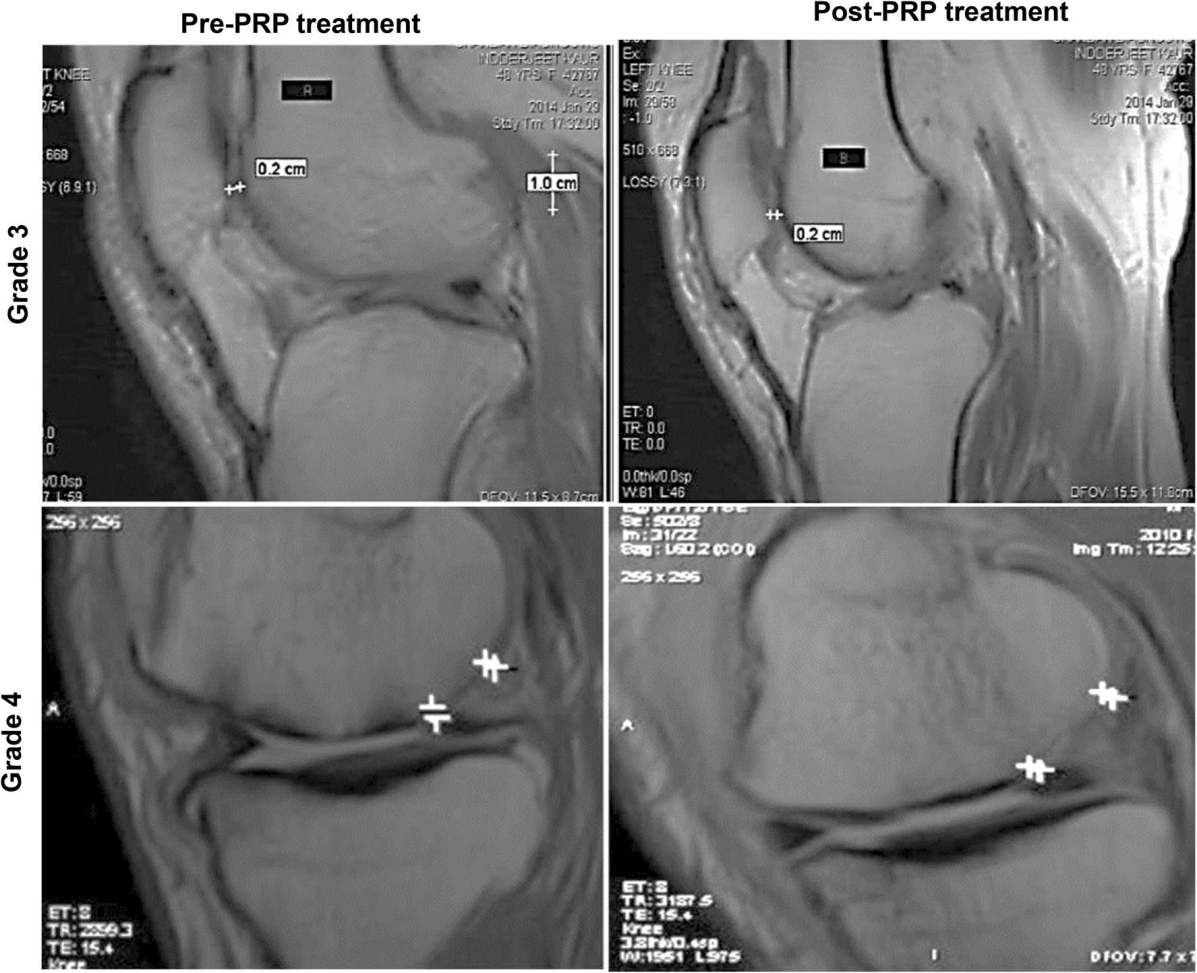
Evaluation of articular cartilage thickness using MRI after one year of PRP treatment in patient with grade 2 and grade 3 OA knee.

The original Article has been corrected.

