# Supplementary material for: Platelet-rich plasma (PRP) in osteoarthritis (OA) knee: Correct dose critical for long term clinical efficacy
Source: Sci Rep. 2021 Feb 17;11:3971. doi: 10.1038/s41598-021-83025-2 (PMC7889864; doi:10.1038/s41598-021-83025-2)
Supplement: Supplementary file 1 — Supplementary Information. [file 41598_2021_83025_MOESM1_ESM.docx]

**Platelet rich plasma (PRP) in Osteoarthritis (OA) knee: Correct dose critical for long term clinical efficacy**

**Himanshu Bansal^1^***, Jerry Leon^2^, Jeremy L. Pont^3^, David A. Wilson^3^, Anupama Bansal^1^, Diwaker Agarwal^4^, Iustin Preoteasa^5^

***Corresponding author:** Himanshu Bansal, Anupam Hospital, Rudrapur, Uttarakhand, India

Email: hbansal@drhbf.org

^1^Anupam Hospital, Rudrapur, Uttarakhand, India

^2^ PMR Advance Health Institute Mayaguez, Puerto Rico, USA

^3^ Pheonix Helse, Norway

^4^ Mercy Medical Centre, Roseburg, Oregon, USA

^5^ Alpha Medica Stem Clinic, Romania

**Email Id:**

Himanshu Bansal hbansal@drhbf.org

Jerry Leon lynnjerry2002@gmail.com

Jeremy L. Pont Jpont7@gmail.com

David A. Wilson dwilson147@gmail.com

Anupama Bansal drbansalh2019@gmail.com

Diwaker Agarwal diwakera@hotmail.com

Iustin Preoteasa Iustin.preoteasa@alphamedica.ro

**Supplementary Tables**

**Supplementary Table 1:** Adverse effect of PRP or HA application

**Supplementary Table 2:** Inclusion criteria and exclusion criteria for patient selection

**Supplementary Table 1:** Adverse effect of PRP or HA application

| **Adverse effect** | **PRP group**  **Total patient (n=64)**  **Number of patient (%)** | **HA group**  **Total patient (n=68)**  **Number of patient (%)** |
| --- | --- | --- |
| Synovitis | 3 (4.6%) | 7 (10.2%) |
| Mild to moderate pain following the injection | 34 (53.1%) | 34 (50 %) |
| Severe pain | 3 (4.6%) | 6 (9.2%) |
| Transient adverse effects such as giddiness, headache, nausea, palpitation, sweating, increased BP, respiration and pulse rates | 20 (31.2%) | 26 (38.2%) |

PRP - Platelet-rich plasma; HA- Hyaluronic acid, n= number of patients

**Supplementary Table 2:** Inclusion criteria and exclusion criteria for patient selection

| **Inclusion criteria** | **Exclusion criteria** |
| --- | --- |
| 1. Symptomatic knee, with pain for at least 3 month or swelling 2. Ability to provide informed consent 3. Analgesics usage at least once a week 4. Less than 30 min of morning stiffness 5. Clinical and radiological criteria of grading scale of osteoarthritis grade 1 and 2 by the American College of Rheumatology | 1. Presence of secondary OA knee due to injury, inflammatory or metabolic rheumatic illness, or osteonecrosis 2. Prior intra-articular infusion of hyaluronic acid (HA), including lavage and corticosteroids within three months 3. Acute osteoarthritic knee (JSW <1 mm) requiring imminent surgery 4. Patients with other systemic co-morbidities (underlying diabetes, blood dyspraxia, inflammatory conditions, pre-existing joint pathology, on immunosuppressant, taking steroids for systemic illness, previous surgical or/ and biological treatments for current injury). |

OA- Osteoarthritis, JSW- Joint Space Width

**Supplementary Table 3:** Levels of proinflammatory cytokine IL6, IL8 and TNF- α in synovial fluid of patients treated with PRP and HA group

| **Mediator** | **Group** | **Baseline (pg/ml)**  **Mean±SD (Range)** | **1 month (pg/ml)Mean± SD (Range)** | **12 month (pg/ml) Mean±SD (Range)** | ***P* value**  **Baseline vs. 1 months** | ***p* value Baseline vs. 1 year** |
| --- | --- | --- | --- | --- | --- | --- |
| **IL-6** | PRP | 163.5±157.6  (7.2–1686.4) | 124.2±117.3  (5.4–1248.3) | 164.9±134.5  (5.1–1272.1) | *P*<0.001 | *P*>0.05 |
|  | HA | 158.2±138.2  (7.6–1527.3) | 148.4±126.6  (7.6–1213.6) | 167.2±145.9  (5.3–1315.3) | *P*<0.001 | *P>0.05* |
| ***P-* value PRP vs. HA** | | *P*> 0.05 | *P*<0.05 | *P*>0.05 |  |  |
| **IL-8** | PRP | 395.2±283.8  (15.1–3323.5) | 392.3±216.3  (12.5–3032.2) | 402.6±362.3  (15.7–3637.7) | *P>0.05* | *P>0.05* |
|  | HA | 389.7±225.2  (14.5–3714.6) | 390.2±268.5  (11.4–3211.6) | 398.1±204.2  (15.7–3837.7) | *P>0.05* | *P >0.05* |
| ***P-* value PRP vs. HA** | | *P*>0.05 | *P*>0.05 | *P*>0.05 |  |  |
| **TNF- α** | PRP | 7.2±4.8 (0.6–14.8) | 5.1±2.7 (0.3–11.7) | 6.8±5.4(0.2–11.2) | *P<*0.001 | *P*>0.05 |
|  | HA | 7.5±4.3 (0.5–13.5) | 6.4±3.6 (0.2–12.6) | 8.1±6.7 (0.3–15.4) | *P*<0.05 | *P>0.05* |
| ***P-* value PRP vs. HA** | | *P*>0.05 | *P*<0.05 | *P*>0.05 |  |  |

PRP - Platelet-rich plasma; HA- Hyaluronic acid, SD- Standard deviation
